# A Short-Term Efficacy of Anlotinib in the Treatment of Refractory Nasopharyngeal Inverted Papilloma: A Case Report

**DOI:** 10.3389/fonc.2021.648895

**Published:** 2021-08-23

**Authors:** Pan Yang, Gang Meng, Qiuxia Shu, Yan Dong, Chong Li, Yujiao Lu, Jianjun Li

**Affiliations:** ^1^Department of Respiratory and Critical Care Medicine, First Affiliated Hospital of Army Medical University, Chongqing, China; ^2^Department of Pathology, First Affiliated Hospital of Army Medical University, Chongqing, China; ^3^Department of Oncology, First Affiliated Hospital of Army Medical University, Chongqing, China; ^4^Genecast Biotechnology Co., Ltd, Chongqing, China

**Keywords:** treat, short-term curative effect, local canceration of refractory nasopharyngeal inverted papilloma, a case report, anlotinib

## Abstract

To our knowledge, no studies have reported the use of anlotinib in the treatment of locally cancerous nasopharyngeal inverted papillomas that cannot be operated on or treated with radiotherapy. Here, we report a case of a 53-year-old woman diagnosed with recurrent local canceration of nasopharynx papilloma. Magnetic resonance imaging (MRI) showed that the right parapharyngeal space, nasopharynx, and ethmoid sinus were changed, and recurrence was considered. There was no indication for surgery or radiotherapy. Imaging showed that the tumor had obvious enhancement and abundant blood vessels. Immunohistochemistry showed that vascular endothelial growth factor receptor (VEGFR) 2 expression was positive in papilloma tissue and in local canceration tissue of the papilloma. After the patient’s consent was obtained, anlotinib treatment was started in May and ended in November 2019. Then, the patient was treated with intensity-modulated radiotherapy (IMRT) with planning gross tumor volume (PGTV) 66 Gy, planning clinical tumor volume 1 (PCTV1) 60 Gy, and planning clinical tumor volume 2 (PCTV2) 54 Gy in 33 fractions. No disease recurrence was reported at 4 months after radiotherapy.

## Introduction

Inverted papilloma (IP) is a benign epithelial tumor of the nasal cavities and paranasal sinuses, and IP of the nasopharynx is rare. IP rates for sinonasal cavity tumors range from 0.4 to 7%. However, three main characteristics make it distinguishable from other sinonasal tumors: its strong invasiveness, high rate of recurrence, and association with carcinoma (1). Studies have shown that nasal inverted papilloma (NIP) has a malignancy rate between 6 and 13%, with most cancers being squamous cell carcinomas and a few being malignant adenocarcinomas (2).

The reason for recurrent papilloma relapse is unknown. Radiotherapy and surgical resection are the main treatments. However, there is no good method for refractory IP. The early symptoms of NIP malignancy are not typical, and some patients do not pay enough attention to the symptoms. Most of the cases diagnosed as malignant lesions are diagnosed too late. When NIP invades the orbit, the base of the skull, brain tissue, and other parts, radiotherapy and surgical procedures become very difficult, resulting in tumors that are not easy to control. Therefore, it is urgent to develop effective and safe treatments for NIP region cancer patients who cannot undergo surgery or radiotherapy. Anlotinib hydrochloride is a new oral tyrosine kinase inhibitor independently developed in China that has the dual effects of inhibiting tumor angiogenesis and tumor growth (3, 4). To the best of our knowledge, this is the first case report of local canceration of IP in the nasopharynx in which anlotinib therapy was effective. Therefore, we suggest that the use of anlotinib should be considered when nasopharynx IP shows local canceration or when nasopharynx IP cannot be treated by surgery or with radiotherapy, as anlotinib may provide a new and appropriate treatment for these patients.

## Case Report

**Figure 1:**
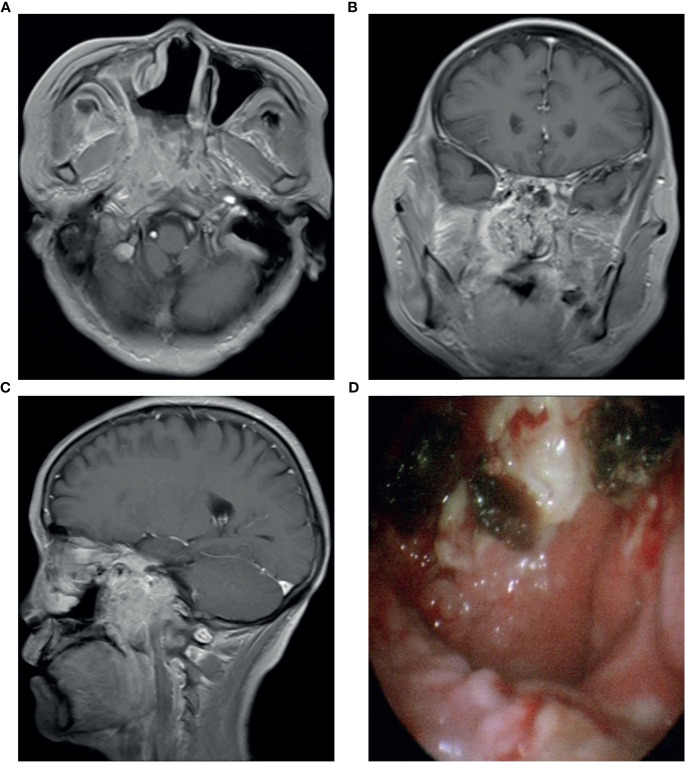
Contrast enhancement T1-weighted MR imaging showed that the tumor invaded the parapharyngeal space and nasopharynx **(A–C)**; **(D)** Electronic nasopharyngoscope showed new organization in the nasopharynx.

**Figure 2:**
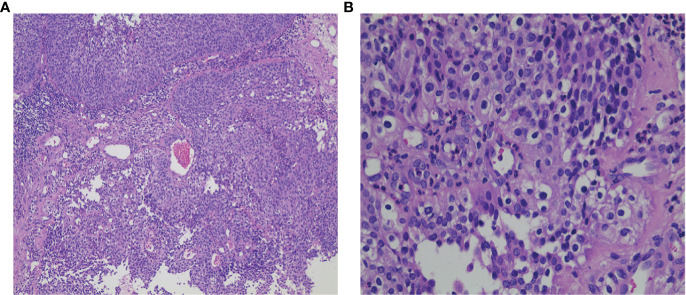
**(A)** H&E staining of local canceration of the inverted papilloma (×100); **(B)** H&E staining of local canceration of the inverted papilloma (×400).

**Figure 3:**
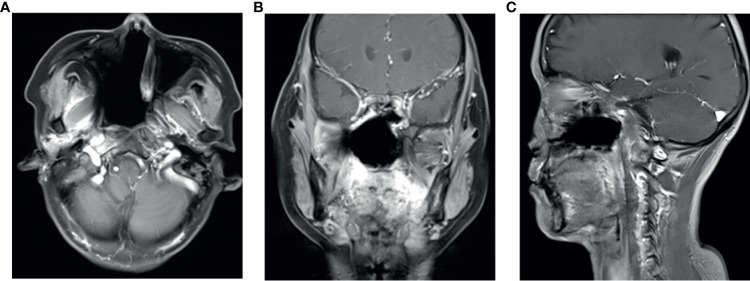
After three cycles of anlotinib treatment, the August 2019 contrast enhancement T1-weighted MR imaging showed the primary tumor was significantly reduced **(A–C)**.

A 53-year-old Chinese woman was admitted to the local hospital for repeated right ear pus with hearing loss in November 2016. The examination showed a new growth in the right external auditory meatus and left nasopharyngeal region. However, the patient did not have obvious nasal congestion, dizziness, headache, or other discomfort. Pathology revealed IPs in the nasopharynx and squamous papillary hyperplasia in the external auditory meatus with focal high-grade intraepithelial neoplasia. No distant metastasis was found on chest abdominal computed tomography (CT) or brain MRI. Surgical resection was performed. Since then, due to recurrence of the lesions in the nasopharynx and right ear, the patient underwent three operations in our hospital, and the postoperative pathology showed IP of the nose and papilloma of the middle ear. During this period, there was sometimes pus or bloody secretion in the right ear. On March 21, 2019, the patient went to our hospital again because of nasal obstruction, headache, and dizziness. MRI of the nasopharynx showed that the right parapharyngeal space, nasopharynx, and ethmoid sinus were changed, and the possibility of recurrence was considered (**Figure 1**). The biopsy showed local canceration of the IP (**Figure 2**). Operation was not suitable. Therefore, 10 mg of anlotinib hydrochloride was given orally daily from June 2019 to November 2019. Two months later, the tumor was significantly reduced (**Figure 3**), and the patient’s nasal obstruction, dizziness, and headache were obviously relieved. Then, nasopharyngeal IMRT was performed from November 2019 to December 2019, with a PGTV of 66 Gy, PCTV1 of 60 Gy, and PCTV2 of 54 Gy. No disease recurrence was reported at 4 months after radiotherapy. The patient denied a chronic history of hypertension or diabetes and any family history of malignant tumors.

## Discussion

Recurrence may be related to incomplete resection, mostly due to bone destruction (5), surgical methods (5), frontal sinus involvement (5, 6), histological features (hyperkeratosis, high mitotic index, severe epidermal hyperplasia) (7), and (human papillomavirus) HPV infection (8). The incidence of local canceration of nasal IP ranges from 5 to 15% (9).

According to the Krouse stage (10), there is no surgical indication in stage T4. Radiation therapy (RT) may be considered for IP in two circumstances: associated carcinoma and impossibility of surgery (11–13). However, chemotherapy research is limited, and fluorouracil-based chemotherapy is a common treatment for local canceration of nasal IP; however, the efficacy of fluorouracil-based chemotherapy is not good. Specialists think that the differentiation degree of squamous cell carcinoma originating from the NIP is insensitive to chemotherapy (7).

Anlotinib is a new small molecule and multitarget tyrosine kinase inhibitor independently developed in China. It can strongly inhibit angiogenesis-related downstream pathways mediated by VEGFR, fibroblast growth factor receptor (FGFR), and platelet-derived growth factor receptor (PDGFR) and interfere with the proliferation and migration of endothelial cells to form lumen, thereby inhibiting the formation of microvessels, and can interfere with multiple biological processes of tumor cells through inhibition of mast/stem cell growth factor receptor **(**c-kit) (14). The inhibitory effect of anlotinib on VEGFR-2 and VEGFR-3 signaling is the strongest, with half maximal inhibitory concentration (IC50) values of 0.2 and 0.7 nmol/L, respectively. Anlotinib is orally administered once a day for 2 weeks and stopped for 1 week, to increase tolerability (15). At present, relevant studies have confirmed that anlotinib is effective for non-small-cell lung cancer (16), colorectal cancer (17), thyroid cancer (18), renal cancer (19), soft tissue sarcoma (20), and other solid tumors, with good safety and controllable adverse reactions. The alter 0303 trial was conducted in 437 non-small-cell lung cancer (NSCLC) patients with stage IIIB/IV who had received at least two previous systemic chemotherapy regimens. They were randomly assigned to receive either anlotinib (n = 294) or placebo (n = 143) until disease progression or intolerable toxicity. The results showed that the median OS (9.6 months *vs* 6.3 months) and PFS (5.4 months *vs* 1.4 months) were significantly prolonged by the single agent. The incidence of adverse events was similar to that of the control group, and the main adverse reactions were hypertension (64.6%), fatigue (46.3%), increased thyrotropin (44.6%), and hand-foot skin reactions (43.2%).

In this case, the local canceration of nasopharynx IP occurred after multiple operations. The recurrence period was short (1–6 months), but there was no viral inclusion body, and HPV infection was not detected by gene sequencing.

**Figure 4:**
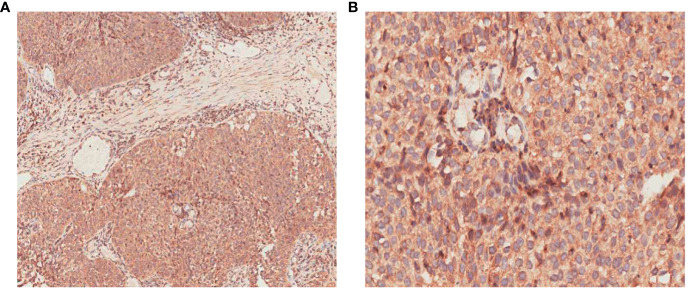
Immunohistochemical staining of local canceration of the inverted papilloma. **(A)** Elevated cytoplasmic expression of VEGFR-2 with color brown in solid nests (×100); **(B)** Elevated cytoplasmic expression of VEGFR-2 with color brown in solid nests (×400).

**Figure 5:**
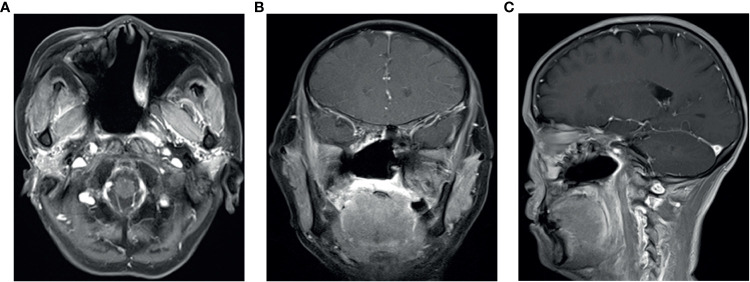
**(A–C)** In April 2020, Nasopharyngeal MRI showed that there was no local recurrence after radiotherapy and anlotinib treatment. Figures **A–C** show the local situation from transverse, coronal and sagittal positions, respectively.

The patient’s primary tumor invaded the skull base, leading to side effects caused by the large target volume of radiotherapy. Chemotherapy was refused. We analyzed the genes in the tissue and found no common sensitive mutation. The immunohistochemistry results showed that the papilloma tissue and papilloma local canceration tissue were positive for VEGFR2 and that the expression intensities were the same (**Figure 4**); however, the expression of VEGFR1, VEGFR3, PDGFR-β, and c-kit was not enhanced in the tissues. Therefore, we chose anlotinib hydrochloride as treatment. The method of administration was 10 mg once per day, with 2-week continuous use and 1 week of rest, and the treatment had good compliance, had no specific side effects, and could be tolerated. After 2 months of treatment, the primary tumor was significantly reduced (**Figure 3**), partial remission (PR) was evaluated, and radiotherapy was then carried out. There was no tumor focus observed in the most recent re-examination, and the duration of tumor disappearance has reached 4 months (**Figure 5**). In fact, our observation period is more than 1 year, but there is a lack of imaging data. Due to personal reasons, the patient did not return to the hospital for re-examination in the later period. We have been following up the patient regularly by telephone. The last follow-up time was July 15, 2021. The patient’s general condition was very good. She was able to engage in general physical activities. The PS score was 0–1. The patient had no nasal congestion, bloody discharge, tinnitus, dizziness, and headache. We will continue to follow up the patient.

VEGFR is highly expressed in many kinds of malignant tumors. However, there are few reports of the high expression of VEGFR2 in papillomas (21). In fact, this is a case of local canceration of papilloma in which the papilloma tissue accounts for the majority the cancer, while the canceration tissue accounts for a small proportion. The expression of VEGFR2 in local canceration tissue is not significantly higher than that in papilloma tissue. However, some studies have shown that the expression of VEGFR2 in nasopharyngeal carcinoma was significantly higher than that in healthy people (22). Zhou et al. (22) compared the expression of VEGFR-2 between nasopharyngeal carcinoma and nasal inflammatory tissue by immunohistochemical method. In 50 cases of nasal inflammatory tissues, VEGFR-2 was moderately expressed in 10 cases (20.00%) and low expressed in 40 cases (80.00%). Among the 50 specimens of nasopharyngeal carcinoma, VEGFR-2 was strong expressed in 26 cases (52.00%), moderately expressed in 20 cases (40.00%), and low expressed in 4 cases (8.00%). The expression of VEGFR-2 in nasopharyngeal carcinoma was significantly higher than that in nasal inflammatory tissue (*P* < 0.05).

This case shows that anlotinib provides a new treatment option for locally cancerous nasopharyngeal IP patients who are unable to undergo surgery or radiotherapy. Future clinical research is needed to determine the proportion of patients with NIP malignant transformation and how extensively this kind of patient is represented among those who are treated with anlotinib.

## Data Availability Statement

The original contributions presented in the study are included in the article/supplementary material. Further inquiries can be directed to the corresponding author.

## Ethics Statement

Ethical review and approval was not required for the study on human participants in accordance with the local legislation and institutional requirements. The patients/participants provided their written informed consent to participate in this study. Written informed consent was obtained from the individual(s) for the publication of any potentially identifiable images or data included in this article.

## Author Contributions

PY: writing the article and analyzing the experimental results. GM, YD: pathological results analysis and image collection. QS, CL, and YL: Follow-up of medical records and immunohistochemical test. JL: plan making, publishing funds, article revision. All authors contributed to the article and approved the submitted version.

## Conflict of Interest

Author YL was employed by company Genecast Biotechnology Co., Ltd.

The remaining authors declare that the research was conducted in the absence of any commercial or financial relationships that could be construed as a potential conflict of interest.

## Publisher’s Note

All claims expressed in this article are solely those of the authors and do not necessarily represent those of their affiliated organizations, or those of the publisher, the editors and the reviewers. Any product that may be evaluated in this article, or claim that may be made by its manufacturer, is not guaranteed or endorsed by the publisher.
